# A monoclonal antibody targeting amyloid β (Aβ) restores complement factor I bioactivity: Potential implications in age-related macular degeneration and Alzheimer’s disease

**DOI:** 10.1371/journal.pone.0195751

**Published:** 2018-05-21

**Authors:** Kameran Lashkari, Gianna Teague, Hong Chen, Yong-Qing Lin, Sanjay Kumar, Megan M. McLaughlin, Francisco J. López

**Affiliations:** 1 Schepens Eye Research Institute, Mass Eye & Ear, Harvard Medical School, Boston, Massachusetts, United States of America; 2 Alliance Pharma, Malvern, Pennsylvania, United States of America; 3 Alternative Discovery & Development, GlaxoSmithKline, King of Prussia, Pennsylvania, United States of America; University of Manchester, UNITED KINGDOM

## Abstract

Activation of the alternative complement cascade has been implicated in the pathogenesis of age related macular degeneration (AMD) and Alzheimer’s disease (AD). Amyloid β (Aβ), a component of drusen, may promote complement activation by inhibiting CFI bioactivity. We determined whether Aβ reduced CFI bioactivity and whether antibodies against Aβ including a monoclonal antibody, GSK933776 could restore CFI bioactivity. We also measured CFI bioactivity in plasma of subjects with AMD and AD. In support of the GSK933776 development program in AMD (geographic atrophy), we developed a quantitative assay to measure CFI bioactivity based on its ability to cleave C3b to iC3b, and repeated it in presence or absence of Aβ and anti-Aβ antibodies. Using this assay, we measured CFI bioactivity in plasma of 194 subjects with AMD, and in samples from subjects with AD that had been treated with GSK933776 as part of the GSK933776 development program in AD. Aβ reduced the CFI bioactivity by 5-fold and pre-incubation with GSK933776 restored CFI bioactivity. In subjects with AMD, plasma CFI levels and bioactivity were not significantly different from non-AMD controls. However, we detected a positive linear trend, suggesting increasing activity with disease severity. In subjects with AD, we observed a 10% and 27% increase in overall CFI bioactivity after treatment with GSK933776 during the second and third dose. Our studies indicate that CFI enzymatic activity can be inhibited by Aβ and be altered in proinflammatory diseases such as AMD and AD, in which deposition of Aβ and activation of the alternative complement cascade are believed to play a key role in the disease process.

## Introduction

Activation of the alternative complement cascade is believed to be involved in the pathogenesis of age-related macular degeneration (AMD), a common cause of central vision loss among individuals over 55 years of age [1, 2]. Early and intermediate stages of AMD are phenotypically defined by deposition of yellowish lipoprotein accumulations between the retinal pigment epithelium (RPE) and the Bruch’s membrane called drusen [3–6]. Proteomics and histochemical studies have demonstrated the presence of inflammatory proteins and lipids within drusen along the Bruch’s membrane [3–6]. These inflammatory proteins are believed to trigger innate immunity through activation of the alternative complement cascade [2, 7]. Genome-wide association studies have revealed genetic variants of different members of the alternative complement cascade as being important in the development of AMD. These include, but are not limited to, complement factor (CF) H, CFI, CFB, complement component C3 [8–11].

**Fig 1:**
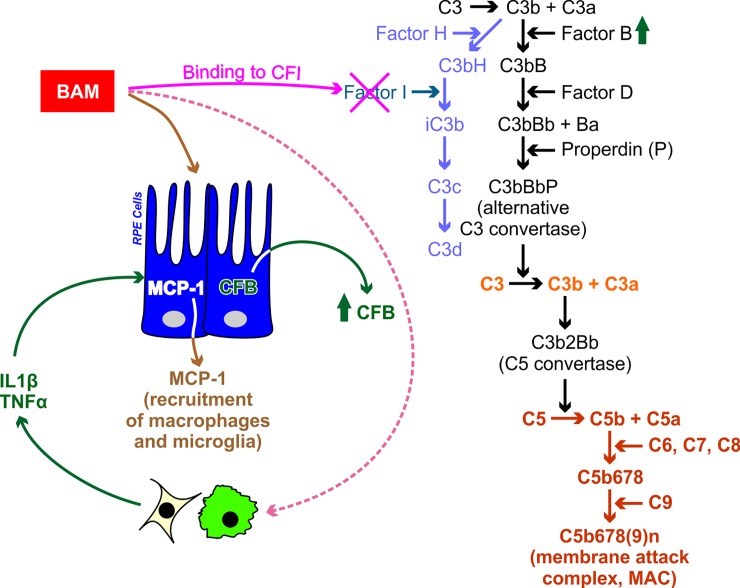
Postulated mechanism of amyloid β (Aβ)-mediated modulation of the alternative complement cascade [19, 20]. Schematic shows the alternative complement pathway. **Magenta text and cross**- interaction of Aβ with complement factor I (CFI) causes reduction of its enzymatic activity and a reduction of the conversion of C3bH to iC3b. **Light Blue text**- Degradation pathway of C3b to C3d via C3bH, modulated by complement factor H (CFH) and CFI. **Black text**- conversion of C3b to C3 convertase. **Orange**-Amplification loop C3 to C3a + C3b. **Red**-Termination phase resulting in conversion of C5 to C5b78(9)n (membrane attack complex [MAC]). Aβ directly and indirectly produces a local inflammatory environment in retinal pigment epithelial (RPE) cells by modulating release of MCP-1, which leads to recruitment of macrophages and microglia locally, and production of TNF-α and IL1β. The action of these factors on the RPE leads to the release of complement factor B (CFB, green), which in turn is the second mechanism leading to activation of the alternative complement cascade [20].

Among the numerous proinflammatory factors discovered in drusen, amyloid β (Aβ) is a notable constituent [12]. Aβ is also present in plaques associated with Alzheimer’s disease (AD) [13, 14]. In AMD, Aβ induces and sustains a local inflammatory milieu and induces release of other proinflammatory and proangiogenic factors that contribute to the inflammatory state [15–17]. It is believed that a major contribution of Aβ to the proinflammatory milieu is through its modulation of CFI bioactivity, one of the key breaks in the alternative complement cascade [18–20]. CFI, together with CFH, tightly control C3 convertase formation through their actions on C3b (Fig 1; light blue). Using *in vitro* and mouse models, Wang et al (2008)[19] showed that in presence of Aβ, CFI enzymatic activity was reduced, leading to decrease in production of the iC3b (Fig 1). This Aβ-induced reduction in CFI bioactivity, in addition to an indirect increase in CFB concentrations, ultimately cooperate to initiate the proinflammatory cascade of the alternative complement cascade, leading to the membrane attack complex [20]. It has been postulated that Aβ sustains maintenance of a low-grade inflammatory state within the subretinal space. In parallel to this, studies on CFH knock out, and APO E knock in mice fed a high-fat diet, have shown deposition of Aβ in the Bruch’s membrane, with subretinal deposits resembling drusen. Interestingly, these studies have shown that antibodies to Aβ reduce the drusen-like deposits by removing Aβ from the subretinal tissue [21, 22].

Given the established relationship between Aβ and CFI in modulating the alternative complement cascade, and in support of the GSK933776 development program in AMD (geographic atrophy), we asked the following questions: (1) Are Aβ peptides (1–40 or 1–42) able to reduce CFI bioactivity to convert C3b to iC3b in an *in vitro* cofactor assay? (2) Do antibodies against Aβ, including a mAb that targets the N-terminal portion of the molecule (GSK933776), restore CFI bioactivity that has been inhibited by Aβ peptides in an *in vitro* assay? (3) Is this relationship quantifiable in a quantitative or pharmacological manner? In addition, the cofactor assay was adapted as a method for quantification of CFI enzymatic activity in human plasma. CFI concentrations and bioactivity, using the newly developed assay, were measured in a cohort of subjects with various stages of AMD. In addition, we also evaluated CFI bioactivity in archived plasma samples of subjects that underwent intravenous (IV) infusions of an anti-Aβ antibody in a phase I trial of AD as a potential measure of target engagement.

## Materials and methods

### Preparation of standards and Aβ solutions

CFI, CFH, C3b, iC3b were purchased from Complement Technology (Tyler, Tx, USA). Ultrapure recombinant Aβ peptides (1–40 and 1–42) were purchased from Covance (Princeton, NJ, USA; www.Covance.com). Anti-Aβ mAb, GSK933776, evaluated in AD phase I trial (BA1106006; NCT00459550), was obtained from GlaxoSmithKline (GSK), King of Prussia, PA (USA).

The reference standard iC3b (1.1 mg/ml) was used to prepare calibration standards at concentrations between 5 ng/ml—10000 ng/ml in assay buffer (10 mM Tris, 60 mM NaCl, 0.1% BSA, 0.1% Tween 20, pH 7.2).

Aβ(1–42) or Aβ(1–40) was suspended in phosphate-buffered saline (PBS) by sonication, adjusted to pH ~ 7 to a final concentration of 1 mM solution. To enhance A**β** inhibitory activity against CFI, the A**β** solution was incubated at 37°C overnight with shaking at 600~1200 rpm in order to oligomerize the peptides. In the absence of this treatment, activity of the A**β** peptides is not present (data not shown). A**β** solutions were stored at 4°C for immediate experiments, and at -70°C for future experiments. A**β** solutions with a half maximal inhibitory concentration (IC_50_) of 1~10 pM was used for most of the experiments.

### iC3b ELISA

During the course of the study, two ELISA methods were used. In the first half of the study, the iC3b kit from Quidel (Santa Clara, CA, USA) was used according to manufacturer’s instructions. This assay uses an antibody directed against a neo-epitope generated after CFI action, therefore, it is specific to iC3b and does not cross-react with C3b. An in-house ELISA method using the same antibody was also developed for measuring iC3b concentrations. Briefly, a 96-well plate was coated with monoclonal anti-human iC3b antibody (Quidel, San Diego, CA, USA) and incubated overnight at 4°C. The plate was then washed 5 times with 300 μl of wash buffer per well using a plate washer, and each well was filled with 200 μl of blocking buffer. The plate was incubated for 1 hour at room temperature (RT) with shaking, and washed again 5 times. One hunded μl of assay buffer-diluted standards and samples were added to the appropriate wells and incubated with shaking for 1 hour at RT. The plate was then washed 5 times. One hunderd μl of HRP-conjugated anti-human C3c (Bio-Rad, Hercules, CA, USA) was added to each well and incubated with shaking for 1 hour at RT. The plate was then washed 5 times. One hunderd μl of Ultra-TMB ELISA Substrate (Thermo Fisher Scientific, Waltham, MA) was added and incubated for 30 min with shaking at RT, and the reaction was stopped by adding 100 μl of 2 M sulfuric acid per well. The absorbance was then measured at 450 nm. Data were processed using Gen 5 TM software (BioTek Instruments, Inc., Winooski, VT, USA).

### ELISA detection of CFI, free Aβ and total bound Aβ

CFI protein concentrations in human plasma were measured using a commercial ELISA kit from USCN Life Science (Wuhan, China), according to manufacturer’s instructions. Initially, 8 human plasma samples were analyzed, four of which were complement grade, i.e, derived from individual donors, and prepared from blood samples immediately after blood was drawn (from Bioreclamation IVT, Baltimore, MD, USA). Purified human serum CFI protein was also included in the assay. Previously banked human plasma samples from subjects with AMD and non-AMD controls and from clinical trials assessing GSK933776 in patients with AD (NCT00459550 GSK study 106006) [23], were subjected to similar ELISA assays for assessment of CFI levels.

Free Aβ (not bound to antibody) and total Aβ (drug bound and free) concentrations were measured in plasma using an immuno-electrochemiluminescence assay as previously described [23]. In Brief, free Aβ fragments were captured with mAb (GSK933776), which was spotted on plates and detected with 4G8 clone mAb (aa18–22; Covance). Total (drug bound and free) Aβ was captured with the 6F6 clone (aa28-35) mAb and detected with mAb 4G8. Total Aβ (amino acids38-4) was also captured with the 6F6 clone mAb (amino acids 28–35) and detected with the 5G5 mAb (amino acids 38–42). The assay uses Aβ-depleted plasma and Aβ from Innogenetics as the reference standard (sensitivity:15.6–78 pg/mL) [23].

### CFI dose and time-course responses

As shown in Fig 1, CFH and CFI control the conversion of C3b to iC3b. An *in vitro* cofactor assay was established in which CFI converts substrate C3b to iC3b in the presence of cofactor CFH. To characterize the activity of CFI in the assay, CFI at 30, 100, 300, and 1000 ng/ml was incubated at 37°C with 80 pg/ml CFH and 80 pg/ml of C3b for 0.5, 1, 2, 4 and 22 hours. Aβ(1–42) or Aβ(1–40) was added in separate studies at concentrations ranging between 1-10pM to the reaction mixture. The concentration of the end product, iC3b, was determined by ELISA as described earlier. Within the incubation period, which varies depending on the matrix from 30 min to 2 hours, iC3b production increased proportionally to the concentration of CFI within the range tested (data not shown). Complete inhibition of CFI bioactivity was achieved by heating the reaction mixture to 60°C for 2 hours.

### Evaluation of anti-Aβ antibody-mediated restoration of CFI bioactivity

For the experiments with anti-Aβ mAb, 0–300 pg/ml anti-Aβ antibody dilutions were pre-incubated with Aβ(1–42) (0–30 pM) at room temperature (RT) for 5 to 30 min, followed by addition of CFI (0–30 pg/ml). After incubation of the anti-Aβ mAb—CFI mixture at 37°C for 30 to 60 min, CFH (80 pg/ml) and C3b (80 pg/ml) were added into the mixture. After incubating for another 30 to 90 min at 37°C, the reaction mixtures were directly diluted into assay buffer. Levels of iC3b were measured using a specific ELISA (Quidel, San Diego, CA, USA).

For plasma assays, a similar approach was used a described above except that the source for CFI was the plasma present in the reaction mix. CFH (80 pg/ml) and C3b (80 pg/ml) were mixed with plasma, diluted 250x in PBS. Due to the high plasma dilution, endogenous concentrations of CFH and other complement components was negligible (see below for details). The diluted plasma was added to microtiter plates as a series of increasing dilutions (2x series) in PBS (BioreclamationIVT, Baltimore, MD, USA), providing progressively increasing concentrations of endogenous CFI to the reaction mixes. The reaction mixtures were incubated at 37°C for 1 to 2 hours. In some assays, plasma samples that had been pre-diluted 10-fold with PBS and then heated at 60°C for 2 hours were included as negative controls at the highest concentration used in the series. After the incubation, the reaction mixture was used for quantification of iC3b according to the procedures described earlier via a specific ELISA. Concentrations of CFI were also measured in the samples to normalize bioactivity to the concentration of CFI present in the sample.

### Establishment of an *in vitro* plasma cofactor assay

In order to measure the activity of CFI in plasma, the effect of endogenous plasma CFH and other complement cascade components had to be minimized. To achieve this, plasma was diluted at least 250-fold in PBS, so that CFH and C3b in the diluted plasma became negligible (< 1 pg/ml) as compared to the exogenous CFH and C3b in the reaction (80 pg/ml for each). Increasing volumes of plasma (2x serial increasing volumes) were added to the reaction mixes as the source of CFI, this effectively provided a titration of CFI levels provided by the different dilution series. In this way, since the cofactor assay described above is a very sensitive assay for CFI, this cofactor in the diluted plasma would still be in the range of detection when it was used as a source of CFI. In these assays, one unit (U) of CFI bioactivity was defined as the amount of CFI or the volume of plasma required to convert 50% C3b to iC3b in the cofactor assay. These values were used to quantify CFI bioactivity in units (U), and these were then corrected by the concentration in μg of CFI protein as measured by an ELISA (USCN Life Sciences).

### Effect of anti-Aβ antibodies on the activity of Aβ in the cofactor assay

To test the effect of anti-Aβ mAb on Aβ-mediated inhibition of CFI bioactivity, several commercially available mouse monoclonal antibodies targeting different epitopes of Aβ were evaluated. These antibodies were added to the plasma cofactor assay (see above) at concentrations between 100–300 pg/ml (data not shown). Human anti-Aβ mAb, GSK933776, was used between 4–500 μg/ml.

### Plasma collection from subjects with AMD

The assays described in this study were established as part of the development program of GSK933776 for the potential treatment of geographic atrophy, the dry form of advanced AMD. Therefore, we were interested in evaluating whether CFI bioactivity changes during the AMD disease continuum. Plasma CFI levels and bioactivity were evaluated in 194 subjects with AMD that were recruited from 2 sources, the observation phase (pre-treatment) of GSK Phase 2 multi-center trial of GSK933776 in adult patients with geographic atrophy (GA) secondary to AMD (NCT01342926, GSK study 114341), and from the practice of one of the authors (KL). For the latter set of samples, subjects were staged as normal (AREDS stage 0), AREDS I, II, III, GA, and neovascular (wet) AMD. Subjects in the wet AMD category were further divided into active (new onset of disease, untreated) and inactive (treated with anti-VEGF therapy and rendered inactive) categories. Plasma collection procedures from each source were approved under separate institutional review boards (IRB) and Ethics Committees, and according to the Declaration of Helsinki. A written informed consent was obtained form each subject. IRB and Ethics Committees included Sterling IRB, Atlanta, GA, USA; Mass Eye & Ear IRB, Boston, MA USA; Western Institutional Review Board, Puyallup, Washington, USA; University of Utah IRB, Salt Lake City, Utah, USA; The Human Research Program, The Committee on Human Research, University of California San Francisco, San Francisco, CA, USA; The Methodist Hospital Research Institute, Institutional Review Board, Houston, TX, USA; University of Pennsylvania, Office of Regulatory Affairs, IRB Committee Number 5, Philadelphia, PA, USA; The Johns Hopkins Medicine IRB, Baltimore, MD, USA; Henry Ford Health System Research Administration IRB, Detroit, MI, USA; The University of Texas Medical Branch at Galveston IRB, Galveston, TX, USA; The University of California San Diego, Human Research Protection Program, La Jolla, CA, USA; University of California, Irvine, Office of Research Administration, Irvine, CA, USA; Health Sciences IRB, University of South California Health Sciences Campus, Los Angeles, CA, USA; Tufts Health Sciences Campus IRB, Boston, MA, USA; New York Eye & Ear Infirmary IRB, New York, NY, USA; University of Miami Human Subjects Research Office, Miami, FL, USA; University of Virginia, IRB for Health Sciences Research, Charlottesville, VA, USA; University of Kansas Medical Center, Human Subjects Committee, Kansas City, KS, USA; Institutional Review Board Servies, Aurora, Ontario, Canada.

Plasma samples were collected in 8 ml citrate buffer plasma collection tubes (BD Biosciences, CPT, or equivalent), promptly centrifuged according to the manufacturer’s instructions, and the plasma component was aliquoted and stored at -80°C. In the observation GSK subgroup, all subjects had been previously diagnosed to have GA associated with AMD, using standard clinical techniques and confirmed by The Wisconsin Reading Center according to the established protocol. In the second subgroup (KL patients), subjects were evaluated with slit-lamp biomicroscopy, fundus imaging (Cannon Digital Fundus Camera), OCT and fundus autofluorescence imaging (both, Spectralis, Heidelberg System, Heidelberg, Germany). Subjects were then staged for AMD using the AREDS classification [24].

### Plasma samples from subjects with AD in a phase I study

Archived plasma samples from subjects with AD from a phase 1 trial (BA1106006; NCT00459550) were used. Details of this study have been previously reported [23]. This study was conducted under Institutional Review Board (IRB) approval from various IRBs and Ethics Committees and according to the Declaration of Helsinki. These included Mass Eye & Ear IRB, Boston, MA, USA; Royal Brisbane & Women’s Hospital Human Research Ethics Committee, Herston, Australia; South Metropolitan Area Health Service Human Research Ethics Committee, Fremantle Hospital and Health Service, Fermantle, Australia; Human Research Committee, Research Ethics Unit, Austin Hospital, Victoria, Australia; Regional komite for medisinsk og helsefaglig forskningsetikk, Sor-Ost A, Blindem, Oslo, Norway; Regionala Etiksprovningsamnden Stokholm, Stokholm, Sweden. Written informed consent was obtained from each subject prior to the performance of any study-specific procedures including these assays. No minors were included in the study.

**Fig 2:**
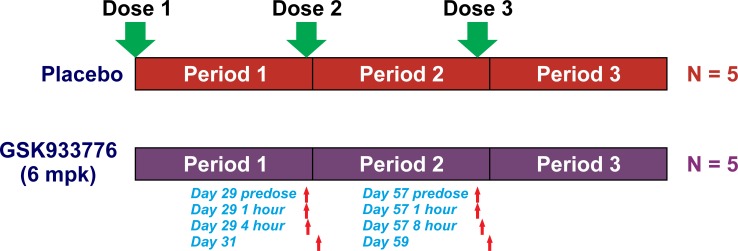
Schematic representation of phase I clinical study of Alzheimer’s disease (AD). Please refer to (BA106006) NCT00459550 and to Andreasen et al. (2011)[23]) for more details of complete study. AD patients received either anti-amyloid β monoclonal antibody, GSK933776 6 mg/kg or placebo. The schematic shows the samples that were tested in the CFI bioactivity assays described in the text. Only samples from days 29–31 (1^st^ and 2^nd^ dose periods), and days 57–59 (2^nd^ and 3^rd^ dose periods) from subjects receiving GSK933776 or placebo were analyzed. A total of 5 subjects per group were assayed. Only 3 samples were available at certain time points. Red arrows indicate samples measured. Reprinted from Andreasen et al. [23] under a CC BY license, with permission from PLOS ONE, original copyright 2015.

Fig 2 shows the schematic of the study design and the samples used to measure CFI bioactivity in our evaluation (yellow shaded areas in the figure). In brief, patients were allocated to either placebo or three doses of GSK933776 (1, 3 and 6 mg/kg; N of 5 per arm). The mAb was administered by IV infusion. Blood was drawn at the beginning of each of the three infusion periods of either placebo or anti-Aβ mAb GSK933776. For our analysis, archived samples from the placebo and 6 mg/kg groups after the second and third infusions were used. The plasma compartment was collected and used for the measurements. Time points for the second infusion (Period 2) included a day 29 pre-dose level, 1 hour, 2 hours and 2 days (day 31) following the second antibody or placebo dose (Fig 2, red arrows). Plasma samples for the third period of infusion, Period 3 included a day 57 pre-dose, 1 hours, 8 hours and 2 days following the third infusion of placebo or antibody. These archived samples would provide an indication as to whether the CFI bioactivity assays could be useful to evaluate the level of target engagement in studies evaluating the potential therapeutic effects of GSK933776. Given the limited number of patients available, the results were evaluated for the direction of the changes after obtaining the weighted average of the samples in each period (area under the curve).

### Statistical analysis of data

Data were analyzed by linear or nonlinear (using a re-parametrized 4-parameter logistic equation [25]), mixed effects models or analysis of covariance (patient’s age used as the covariate), with between group multiple comparisons using the Tukey’s multiplicity correction. Data were analyzed after Box-Cox transformations when appropriate to improve statistical properties of the data [26, 27] using R [28] under Rstudio. Details on the specific analysis and data presentation are included in the legends to the figures.

Data for this study have been deposited at https://www.protocols.io/private/a825dc15eb3d76e02bece56025437376

## Results

### Aβ reduces CFI bioactivity

**Fig 3:**
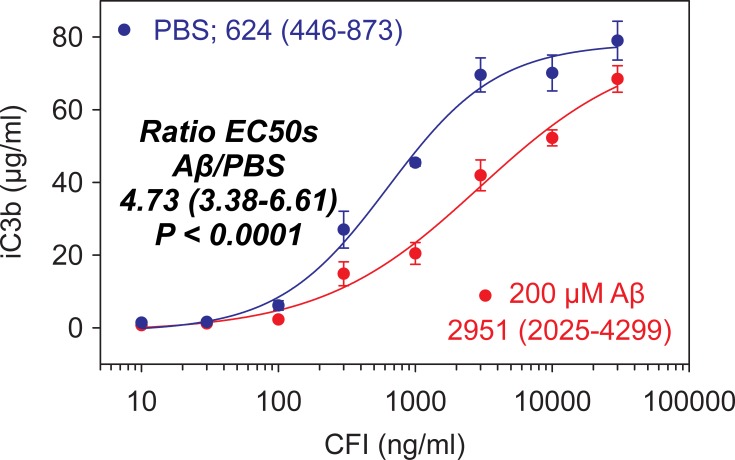
Preincubation of CFI with amyloid β (1–42) (Aβ) reduces CFI bioactivity. CFI (10–30 000 ng/ml) was incubated with Aβ (200 μM) for 1 hour at room temperature. CFH (10 μg/ml) and C3b (80 μg/ml) were added to the reaction and incubated at 37°C for 30 min. Aliquots of the reaction mixture were analyzed for iC3b by ELISA. Pre-incubation with Aβ causes a statistically significant shift of the curve to the right and approximately, a 5-fold decrease in CFI bioactivity. Data are presented as means ± SEM in triplicate wells. A non-linear mixed effects model was used to estimate the ratio of EC_50_ for the dose response curves using a reparametrized 4 parameter logistic equation [25]. Number in parentheses indicate 95% confidence intervals for the estimated EC_50_ ratio or EC_50_ in ng/ml.

In an *in vitro* assay, Aβ(1–42) reduced the enzymatic activity of CFI by approximately 5-fold, in a dose-dependent manner. Fig 3 shows a typical dose curve for iC3b and CFI in presence or absence of Aβ(1–42). Addition of Aβ(1–42) shifts the reaction curve (blue) to the right (red). The ratio of half maximal effective concentration (EC_50_) for Aβ(1–42) over PBS was estimated at 4.73 ng/ml (95% CI, 3.38–6.61) using non-linear mixed effects models, and this difference was statistically significant (P < 0.001). Half maximal effective concentrations for reaction curves in the absence or presence of Aβ(1–42) were 624 ng/ml (95% CI, 446–8,730) and 2,951 ng/ml (95% CI 95%, 2,025–4,299), respectively. The data imply that in the presence of Aβ more C3b will be available to produce C3bH, and the overall reaction cascade will shift to an increased inflammatory potential through production of C3 convertase and C5 convertase (Fig 1). Additional studies were conducted to evaluate the relative ability of Aβ(1–40) or Aβ(1–42) to inhibit CFI bioactivity. The results indicate that Aβ(1–40) exhibited 4.29 (99% CI, 3.29–5.62)-fold more inhibitory activity than Aβ(1–42) under the assay conditions (data not shown).

### Anti-Aβ mAb restores CFI bioactivity that has been reduced by pre-exposure to Aβ

**Fig 4:**
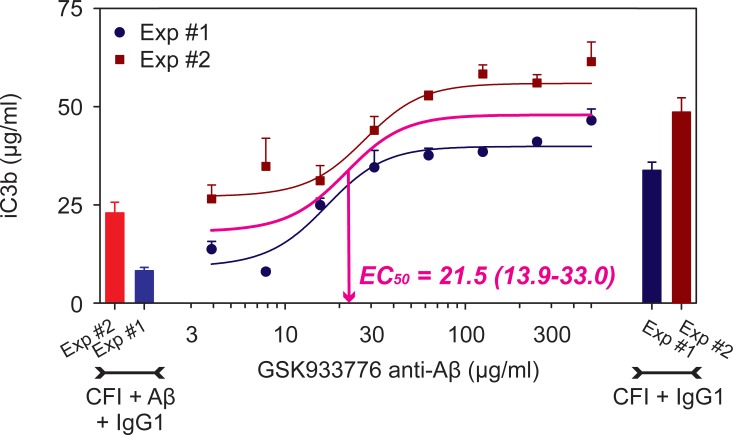
Anti-amyloid β (Aβ) antibody, GSK933776, effectively blocks Aβ’s ability to inhibit CFI bioactivity. Increasing doses of the antibody were preincubated with Aβ (30 μM) for 10 min at room temperature. CFI (1 μg/ml was added and incubated for 20 min at 37°C. CFH and C3b (80 μg/ml) were added and the mixture was incubated for an additional 30 min. Aliquots of the reaction mixture were analyzed for iC3b using ELISA. The curve in magenta shows the global fit of the two curves. The right dark red and dark blue bars show the effect of CFI + IgG1 (non-specific antibody control) on production of iC3b. The left 2 bars in lighter colors show the effect of CFI and IgG1 in presence of Aβ, i.e., reduction in CFI bioactivity. GSK933776 reduced Aβ’s ability to inhibit CFI in a dose-dependent manner (EC_50_ ~ 20 μg/ml). Data are presented as means ± SEM of triplicate determinations for two experiments conducted over 2 separate weeks. A non-linear mixed effects model was used to estimate the EC_50_ of the dose response curves using a reparametrized 4 parameter logistic equation [25]. Numbers in parentheses indicate 95% confidence intervals for the estimated EC_50_.

In the same *in vitro* experimental setting as detailed in previous section, addition of anti-Aβ mAb blocked the inhibitory effects of Aβ on CFI bioactivity, hence restoring the ability of CFI to convert C3b into iC3b (Fig 4). The graph in Fig 4 shows 2 distinct independent experiments conducted in triplicate (shown as red and blue graphs). Analysis of pooled absolute data indicated that the median coefficient of variation across the two studies and dose-response curves was 21.65%. Using a non-linear mixed effects model with a reparametrized 4-parameter logistic equation[25], the global fit of these experiments is shown in the middle curve (magenta in Fig 4). Addition of CFI and IgG1, as a control for anti-Aβ mAb, results in increases in the concentration of iC3b as a result of CFI bioactivity (right side bars in Fig 4). Incubation of CFI and IgG1 with Aβ (1–42) reduces CFI bioactivity as shown in the lighter red and blue bars in Fig 4 (left side bars). GSK933776 restored CFI bioactivity that had been inhibited by the addition of Aβ (Fig 4, symbols and lines in the plot). The estimated EC_50_ was 21.5 μg/ml (95% CI, 13.9–33.0, a precision of the estimate of approximately 20 units, that is less than half of one logarithm for the combination of the two studies). These data indicate that GSK933776 is able to block the ability of Aβ‘s to inhibit CFI bioactivity in this *in vitro* assay and restore CFI bioactivity to normal levels.

### Measurement of CFI bioactivity in plasma samples

**Fig 5:**
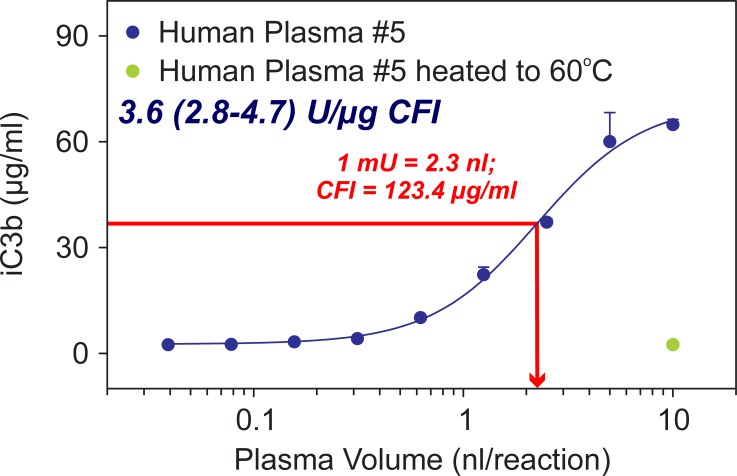
Measurement of CFI bioactivity. CFI bioactivity can be measured in plasma samples using an adapted assay that uses diluted plasma as the source of CFI. CFI bioactivity is temperature sensitive as heating the samples to 60° C abolishes the ability of the sample to generate iC3b (green symbol). Under the configuration of the assay, one can estimate the concentration of plasma that achieve half the amount of iC3b (i.e., 2.3 nl). We define this volume as 1 mU of CFI bioactivity. CFI bioactivity can be measured by determining the concentration of CFI protein in the same sample with an ELISA and correct the activity values by the amount of protein present in the sample, hence CFI bioactivity per unit of protein.

Using the methodology described in Fig 3, we developed a method to measure CFI bioactivity in plasma samples. The CFI bioassay allows the determination of the volume necessary to elicit conversion of half the amount of C3b into iC3b (EC_50_). Fig 5 shows an example of the assay for a human plasma sample (plasma sample #5). Under the configuration of the assay, we can estimate the volume of plasma that would generate half the amount of iC3b (i.e., 2.3 nl in Fig 5). We defined this volume as one mU of CFI bioactivity. Since we can also measure the concentration of CFI protein in the same sample with an ELISA, we can also correct the activity values by the amount of protein present in the sample, and hence measure CFI bioactivity per unit of protein. In this sample, the concentration of CFI was 123.4 μg/ml and, hence, CFI bioactivity in this sample was 3.6 (2.8–4.7) Units/μg CFI protein. In addition, the maximum volume of plasma evaluated in the assay was also tested after heating at 60°C to denature the CFI protein. As shown in Fig 5 (green symbol on the right bottom corner), CFI bioactivity was absent after heating the sample at 60^o^ C for 2 hours, confirming that the structural integrity of CFI is required to elicit conversion of C3b into iC3b.

### Measurements of CFI levels and bioactivity in plasma of subjects with AMD

The CFI bioactivity assay described in this manuscript was developed in support of the development program for GSK933776 for the potential treatment of geographic atrophy. Therefore, CFI protein concentrations and bioactivity levels were measured in plasma samples of a cohort of 194 subjects with AMD (136 female: 58 male). The mean age for these subjects was 78.4 ± 7.6 years. Subjects were staged as normal (AREDS stage 0; N = 33), AREDS I (N = 13), II (N = 4), III (N = 24), GA (N = 37), and wet AMD (N = 83). This order was also used in the linear trend analysis with the idea that disease severity increases with the stage of disease. Subjects in the wet AMD category were further divided into active (new onset of disease, untreated; N = 47) and inactive (treated with anti-VEGF therapy and rendered inactive; N = 36) disease categories.

**Fig 6:**
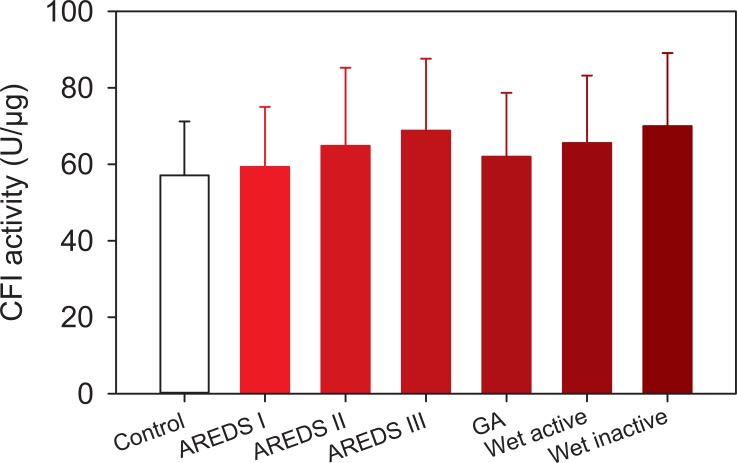
CFI bioactivity in a cohort of subjects with staged AMD and non-AMD normals. Subjects with AMD were staged according to the AREDS classification [24]. CFI bioactivity was determined by measuring the conversion of C3b to iC3b using ELISA for iC3b. Plasma dilutions (0.01–25 μl) were exposed to 80 μg/ml of each CFH and C3b and incubated at 37°C for 2 hours. Levels of iC3b were measured using ELISA. Samples from the same individual were run in the same assay as singlets. To control for the multiple assays conducted, each assay included a control sample. Data were fitted to a 4-parameter logistic equation and EC_50_s estimated. These values were converted to bioactivity in U/ μ g of CFI (1 mU = 1 / EC_50_ in pg/ml). The logarithm of the bioactivity for the experimental samples and control plasma samples were analyzed by a mixed-model ANCOVA, using the control sample as a covariate to correct for inter-run variability. Data are shown as the fitted means in the original scale ± SE estimated from the fitted values using the Taylor series expansion. N = 4 to 47 patients per group. Legends: AREDS, Age-Related Eye Disease Study stages I through III, GA, geographic atrophy, Wet-Inactive, wet active AMD.

Table 1 shows the age distribution of subjects in each of the AREDS categories for this study. Subjects in normal and AREDS I groups were on average younger than subjects in AREDS III and above (P<0.05 for each group compared to normal and/or AREDS I). Levels of circulating CFI were measured with an ELISA (Table 2), and these values were used to correct the observed CFI bioactivity levels. There were no statistically significant differences among the groups independent of age, indicating that systemic CFI levels do not change in AMD. In addition, CFI bioactivity was measured using the same assay as previously described (see Fig 5). A significant increasing linear trend was observed with disease progression (P = 0.0185); however, there were no statistically significant changes in a group to group comparison (Fig 6).

**Table 1:** Age distribution of subjects staged for AMD^§^.

Group	Age Mean ± SE (years)
Normal	72.42 ± 1.21
AREDS Stage I	72.46 ± 1.93
AREDS Stage II	79.00 ± 3.48
AREDS Stage III	79.38 ± 1.42*
Geographic Atrophy	79.65 ± 1.14**
Neovascular AMD (inactive)	81.06 ± 1.16**
Neovascular AMD (active)	80.55 ± 1.02**

^**§**^Please refer to text for details. Data were analyzed by analysis of variance followed by the Tukey test on raw data.

* P< 0.05 difference between the indicated AMD stage patients and normal

** P<0.05 difference between the indicated AMD stage patients and normal as well as AREDS Stage I patients

**Table 2:** CFI levels in a cohort of subjects with AMD and non-AMD normal measured by ELISA*.

Group	CFI levels (μg/ml) Geometric Means ± SE
Normal	25.90 ± 0.97
AREDS Stage I	24.09 ± 1.38
AREDS Stage II	21.50 ± 2.17
AREDS Stage III	24.19 ± 1.00
Geographic Atrophy	25.60 ± 0.85
Neovascular AMD (inactive)	22.53 ± 0.77
Neovascular AMD (active)	23.85 ± 0.71
No significant differences were detected among the groups.	

*Data were analyzed by analysis of covariance using age as the covariate on the logarithm of the data followed by the Tukey test. Results are presented as the geometric means ± SE estimated using the Taylor series expansion.

### Measurement of CFI bioactivity in plasma after treatment with GSK933776 in subjects with AD

**Fig 7:**
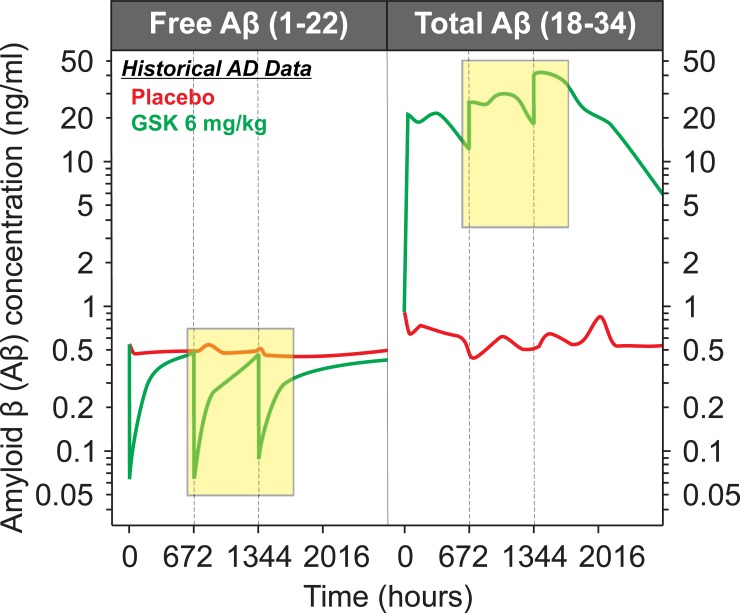
Plasma levels of free and total amyloid β (1–42) (Aβ) in Alzheimer’s disease (AD). Plasma samples were collected from the phase I study of AD, receiving anti-Aβ antibody GSK933776 at 6 mg/kg (see Fig 2). Yellow transparent boxes indicate time intervals where CFI bioactivity measurements were performed. Following 3 doses of GSK933776, total Aβ levels do not appear to have reached steady state. Adapted from Andreasen et al., 2015, Fig 3A, p10 [23].

**Fig 8:**
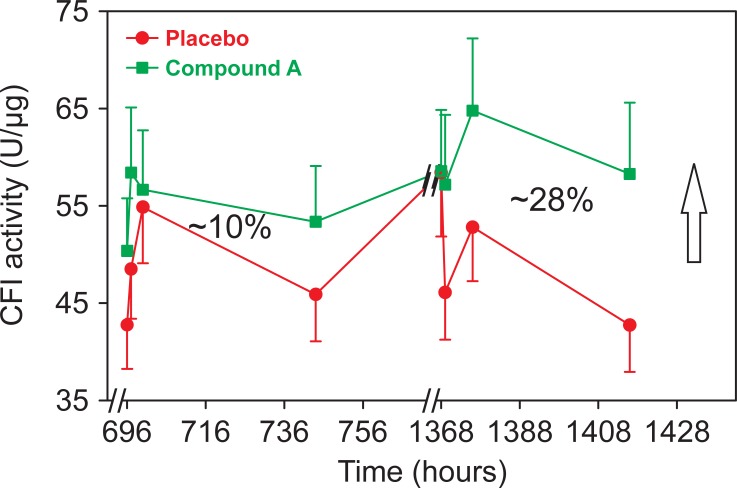
CFI bioactivity in plasma samples of Alzheimer’s disease (AD). Plasma was collected from the phase I study of AD receiving anti-amyloid β antibody GSK933776 at 6 mg/Kg. CFI bioactivity was determined by measuring the conversion of C3b to iC3b using ELISA for iC3b. Plasma dilutions (0.01–25 nl) were exposed to CFI and CFH (80 μg/ml each) and incubated for 2 hours at 37°C. Samples from same individuals were run in singlets within the same assay. Each assay included a control sample. Data were fitted using a non-linear mixed model that used a reparametrized[25] 4-parameter logistic equation and EC_50_s were estimated. The values were converted to bioactivity of CFI-Units/μg of CFI (1mU = 1/EC_50_ in pg). The logarithm of CFI bioactivity for study and control samples were analyzed by a non-linear mixed effects model using the control sample as a covariate to correct for inter-run variability. Data are presented as the geometric means ± SE estimated using the Taylor series expansion (n = 3–5 per group). Percentages shown in the graph were calculated from the integrated areas under the curve for each dosing period using the trapezoidal rule on the geometric means.

Fig 7 shows levels of free (unbound) Aβ and total (bound to the antibody and unbound) Aβ following infusions of placebo or anti-Aβ mAb GSK933776 at 6 mg/kg. The yellow boxes indicate time points available, and in which CFI bioactivity was measured in this study. It is interesting that the levels of total Aβ, indicating the amount of drug present, are not at steady state as levels appear to increase after subsequent doses. In fact, the levels of total Aβ after the third infusion appear to be progressively higher when compared to the previous dose. Fig 8 shows CFI bioactivity in archived plasma samples of subjects receiving either placebo (red line) or 6 mg/kg of anti-Aβ mAb, GSK933776. In the second (696–756 hours) and third (1368–1428 hours) dosing periods, overall increases in CFI bioactivity of ~10% and ~27% were detected, respectively. These differences were not statistically significant in a mixed effect model due to the limited number of samples. However, the increase between the second and third dose mirrored the progressively increasing levels of total Aβ concentrations (which represent the levels of antibody) observed in these samples (see yellow box in the right panel of Fig 7).

## Discussion

CFI plays a key role in the regulation of all complement cascades, alternative, classical and lectin, by channeling C3-derived C3b toward production of iC3b in the breakdown pathway (Fig 1). CFI bioactivity reduces the stores of C3 convertase for complement activation and ultimately reduces production of the membrane attack complex (MAC) [19, 29]. Wang et al [19] have previously shown that CFI binds to and co-precipitates with Aβ. Furthermore, they showed that C3b degradation is inhibited when CFI is preincubated with Aβ [19]. In the current study, we developed a method to specifically address the effects of Aβ on CFI bioactivity in a quantitative *in vitro* assay. Preincubation of CFI with Aβ reduces production of iC3b shifting the CFI dose response curve to the right (i.e., more CFI is required in the presence of Aβ to achieve half maximal concentration of iC3b). This reaction produces a sigmoid curve when iC3b is plotted against CFI with an EC_50_ of 2,951 μg/ml (95% CL = 2,025–4,299). The curve is significantly shifted to the right in presence of Aβ by approximately 5 fold (4.73 μg/ml; [95% CL = 3.38–6.61]), demonstrating that Aβ inhibits CFI bioactivity *in vitro*, and that this effect can be quantitatively measured. We also showed that pre-incubation of Aβ with various anti-Aβ antibodies (data not shown) including a mAb that was dosed in humans GSK933776 (Fig 4), resulted in a dose-dependent restoration of CFI bioactivity (EC_50_ for GSK933776 = 21.5 μg/ml [95% CL = 13.9–33.0]). It has been shown that while both factors co-precipitate with Aβ, the inhibitory effect of Aβ is related to CFI and not to CFH [19]. Wang et al [19] used western blotting, which is not suitable for easy quantification, whereas our assay seems is be able to provide full and objective quantification of these effects *in vitro*.

The inhibitory effect of Aβ on CFI is an indication for the potential role of Aβ as in indirect activator of the complement pathway. It is well documented that Aβ is a key component of drusen associated with AMD [30, 31]. Drusen contain Aβ in various forms, ranging from free peptides to oligomers and fibers. Drusen amyloid vesicles are believed to harbor fibrillar amyloid [31]. These materials can account for local inflammation, as most components of the alternative complement pathway are produced by the RPE monolayer, and do not require any systemic source such as the liver [2]. Chronic local inflammation can result in RPE stress, and progressive loss of outer retinal segments and choriocapillaris, as production of survival/trophic factors such as vascular endothelial growth factor (VEGF), pigment epithelium derived factor (PEDF), and others is altered [32, 33]. In support of these findings, Ding et al [22] and Catchpole et al [21] have independently shown that administration of neutralizing antibodies to Aβ reduced deposition of Aβ in mouse models. Catchpole et al [21] using CFH knock out mice, which is associated with deposition of Aβ around the RPE and Bruch’s membrane, showed that both prophylactic and therapeutic administration of Aβ antibodies reduced deposition of Aβ and C3 in the retina. In a different model, an *APOE4* knock in mice fed a high fat and cholesterol-enriched diet, Ding et al [22] showed deposition of Aβ and complement proteins as basal deposits beneath the RPE. Administration of a different anti-Aβ antibody (against the C-terminus) resulted in reduction of deposits and restoration of electroretinogram. These studies indicate that removing Aβ by systemic antibody administration improves the anatomy of the RPE and Bruch’s membrane layers and may reduce the local inflammatory milieu. It is possible that besides removing Aβ from tissues and circulation, anti-Aβ antibodies reduce the Aβ that is available to locally inhibit CFI bioactivity and reduce breakdown of C3 toward production of membrane attack complex.

Since these studies were designed to support the development program of GSK933776 in geographic atrophy, we also used the CFI bioactivity assay to assess whether concentrations and, more importantly, CFI bioactivity changes throughout the AMD disease continuum using a cohort of subjects with various stages of AMD. Subjects included normal (no-AMD) and AMD, based on AREDS criteria [24]. These included advanced stages of GA, acute wet and treated wet (inactive) AMD. No significant differences were detected in CFI plasma concentrations across these subgroups (Table 2), consistent with previous studies [10]. We subjected these plasma samples to the same *in vitro* assays to quantify CFI bioactivity. We detected no statistical significant differences in CFI bioactivity across these groups. However, we observed a statistically significant linear trend across the disease severity continuum, suggesting progressive increases in systemic CFI bioactivity with disease progression. Recently, in a 2 independent cohort study in patients with AMD [34], we have shown that plasma levels of Aβ(1–40) were elevated in patients with AREDS III and GA. In the first cohort, elevations of 8% and 30% for the AREDS III and GA patients were observed as compared to controls subjects, respectively. In the second cohort of AREDS III and GA, plasma levels were elevated by 0% and 48%, respectively. In the second cohorts, mean ± SE plasma levels were 50 ± 1.30 pg/ml for control, 60 ± 1.35 pg/ml for AREDS III and 159.81 ± 1.42 pg/ml for GA (P < 0.05). Interestingly, plasma levels of Aβ(1–42) were not significantly elevated in either cohort [34]. Although we observed elevated plasma levels of Aβ(1–40), the concentrations present in systemic circulation may not be sufficiently high to reduce CFI bioactivity. Since concentrations at the level of the Bruch’s membrane appear higher than those observed in plasma, while the changes observed systemically are a reflection of what is occurring at the tissue level. These observations therefore suggest that the pro-inflammatory interactions of Aβ on CFI may be occurring on a local level within the subretinal space-RPE and Bruch’s membrane, rather than affecting systemic sources of CFI, despite systemic levels of Aβ. It is possible that this mechanism (Aβ-dependent inhibition of CFI bioactivity) is more efficient locally, and hence contribute to disease pathogenesis at the level of the RPE/Bruch’s membrane interphase. Local sources of complement and related pro-inflammatory proteins are generated by the RPE without the need for systemic supplementation [35, 36]. Follow-up studies would need to evaluate Aβ and CFI levels and bioactivity directly in ocular tissue of AMD patients to determine whether higher local concentrations of Aβ are potentially responsible for a local reduction in CFI bioactivity. Based on these observations, these *in vitro* data recapitulate the role of Aβ in inhibiting CFI bioactivity, and therefore induce a push towards activation of the complement system and formation of MAC locally in the eye.

Using the same methodology, we also studied the effects of anti-Aβ mAb, GSK933776, on CFI bioactivity in a few achieved samples from subjects from phase 1 trial of anti-Aβ systemic therapy for AD. Plasma samples were collected around the second and third infusion periods (see Fig 2). Interestingly, during infusion period, the concentrations of free amyloid initially dipped (due to clearance from the antibody), but returned to placebo-treated levels after termination of each infusion period and prior to the next infusion (Fig 5, left panel). The concentration of total Aβ (representing the antibody load) increased over the infusion periods, but never reached a plateau over the 3 infusion periods studied (Fig 5, right panel). In humans, anti-Aβ antibody treatment may mobilize multiple reservoirs that were not detected or reported by the aforementioned animal studies. Interestingly, the monoclonal anti-Aβ mAb, GSK933776, was able to increase CFI bioactivity by approximately 10% during second infusion and by approximately 27% in the third infusion (Fig 6), an increase consistent with the increasing levels of total antibody concentrations after the third injection. These findings were not significant using a nonlinear mixed effects model given the limited number (3–5) of patient samples available, but suggest that overall GSK933776 enhances CFI bioactivity *in vivo*.

The *in vitro* assays described in this study have allowed efficient and accurate quantification of CFI bioactivity. The methodology could be applied to RPE cells and to determine whether or not RPE-generated CFI is adversely affected by local Aβ deposits. Interestingly, our clinical data show that in AMD, systemic CFI may not robustly engage in pro-inflammatory signaling of the alternative complement pathway, and perhaps these pathways are more robust directly at the ocular tissue level where Aβ concentrations are higher. Recent reports indicating that CFI polymorphisms are associated with AMD [10, 11, 37] support this notion. Further studies evaluating direct measurement of CFI bioactivity at the level of the RPE from AMD eyes would further elucidate this question.
